# Enhanced Detection of Algal Leaf Spot, Tea Brown Blight, and Tea Grey Blight Diseases Using YOLOv5 Bi-HIC Model with Instance and Context Information

**DOI:** 10.3390/plants14203219

**Published:** 2025-10-20

**Authors:** Quoc-Hung Phan, Bryan Setyawan, The-Phong Duong, Fa-Ta Tsai

**Affiliations:** 1 Department of Mechanical Engineering, National United University, Miaoli 360302, Taiwan; andresetyawan180@gmail.com; 2 Department of Mechanical Engineering, HCMC University of Technology and Education, Ho Chi Minh City 7000, Vietnam; phongdt@hcmute.edu.vn

**Keywords:** tea leaf diseases, YOLOv5, convolutional neural network

## Abstract

Tea is one of the most consumed beverages in the world. However, tea plants are often susceptible to various diseases, especially leaf diseases. Currently, most tea farms identify leaf diseases through manual inspection. Due to its time-consuming and resource-intensive nature, manual inspection is impractical for large-scale applications. This study proposes a novel convolutional neural network model designated as YOLOv5 Bi-HIC for detecting tea leaf diseases, including algal leaf spot, tea brown blight, and tea grey blight. The model enhances the conventional YOLOv5 object detection model by incorporating instance and context information to improve the detection performance. A total of 1091 raw images of tea leaves affected by algal leaf spots, tea brown blight, and tea grey blight were captured at Wenhua Tea Farm, Miaoli City, Taiwan. The results indicate that the proposed model achieves precision, recall, F1 Score, and mAP values of 0.977, 0.943, 0.968, and 0.96, respectively, during training. Furthermore, it exhibits a detection confidence score of 0.94, 0.98, and 0.92 for algal leaf spot, tea brown blight, and tea grey blight, respectively. Overall, the results indicate that YOLOv5 Bi-HIC provides an accurate approach for real-time detection of leaf diseases and can serve as a valuable tool for timely intervention and management in tea plantations.

## 1. Introduction

Tea is one of the most consumed beverages in the world, particularly in Asian countries such as Taiwan, Japan, China, and Indonesia, where it is regarded as a daily staple. Furthermore, in many Asian countries, tea is one of the mainstays of the national economy [1]. However, tea plants are susceptible to various diseases that can manifest in different forms and attack different parts of the plant [2,3,4]. Among these diseases, leaf diseases have a particularly serious effect on the yield and quality of the tea. Therefore, timely identification of the onset and tea leaf disease is essential for taking effective preventive measures to improve the quality of the tea, maintain the harvesting yield, and support economic growth [5]. Currently, most tea farms identify tea leaf diseases through manual in situ inspection, followed by detailed disease analysis if required. Hu et al. [6] proposed a visual inspection method for detecting tea leaf blight severity estimation in RGB images obtained under natural scenes. Zou et al. [7] used filter-disc DNA extraction to detect Colletotrichum siamense from infected tea plants rapidly. Manual methods such as those in [6,7] provide a simple and effective means for detecting plant diseases. However, they are time-consuming and resource-intensive, making them impractical for large-scale applications. Accordingly, there is a pressing need for more scalable approaches for the detection and identification of different tea leaf diseases.

Convolutional neural networks (CNNs) are a standard deep learning model in the computer vision field and have been widely used in agriculture in recent years, especially for tea leaf disease detection [3,8,9]. One of the most common CNN models is YOLO (You Only Look Once) [10]. YOLO models perform object detection in images and videos by dividing the input image into grids and making predictions at the grid level. Since YOLO generates bounding boxes and class probabilities within each grid, it is speedy and efficient and is thus well-suited for real-time applications. YOLO has undergone numerous enhancements since its introduction [11,12,13], with each version building on the successes and lessons of its predecessors [14,15,16]. YOLOv1 was first introduced in 2015. Its accuracy and performance were improved continuously through the versions Yolov2 to Yolov4. In 2020, the Yolov5 was introduced and impressed all developers, engineers, and researchers with its faster speed, smaller model sizes, and ease for developers. Lin et al. [17] developed TSBA-YOLO based on the original Yolo-v5 for tea disease detection with an accuracy of 91%. Xue et al. [18] proposed the YOLO-Tea model based on Yolov5 for detecting four different tea leaf diseases, with an increment in accuracy up to 3.5% compared to Yolov5. Bao et al. [19] proposed DDMA-YOLO based on Yolov5 for Tea leaf blight detection, with an increment in the recall up to 6.5% compared to its original Yolov5 model. Yolov7 was introduced in 2022 with improvements for advanced model scaling and an improved backbone design for real-time object detection. Soeb et al. [20] used Yolov7 to detect five different tea leaf diseases with an accuracy of 97%. Yolov8 was introduced in 2023 with improved accuracy and speed for real-time object detection. Ye et al. [21] proposed the Yolov8 modified model for detecting fine-grained disease images with an accuracy of up to 95.26%. Zhan et al. [22] improved a YOLOv8-based BHC target detection model for seven tea leaf diseases with an accuracy reach 94.5%. Yolov9, Yolov10, and Yolov11 are continuously introduced in 2024. YOLOv9 is focused on transformer-based feature extraction and multi-scale detection for accurate training. YOLOv10 is a quantization-aware training and hardware-friendly design for edge AI applications. YOLO11 is an Architectural enhancement with hybrid CNN-transformer models. Islam et al. [23] proposed Yolov10 for tea leaf disease detection with an accuracy of 97.9%. The studies described in [18,19,20,21,22,23] confirmed the feasibility of Yolo models for tea leaf disease detection. YOLOv5 is the most common model and attracts developers with its fast speed, smaller scale model, and easier use.

In this study, a modified YOLOv5 model was developed for tea leaf disease detection called YOLOv5-Bi-HIC. The proposed model comprises four modules in addition to the YOLOv5 backbone: a Bidirectional Feature Pyramid Network (Bi-FPN), Small Object Detection Head (SODH), Involution, and Convolutional Block Attention Module (CBAM). By integrating these modules, YOLOv5 Bi-HIC aims to achieve more focused, accurate, and reliable object detection results, particularly for small objects and those in cluttered, complex backgrounds such as tea plants in natural settings. The proposed model is then used to detect tea leaf diseases, namely algal leaf spot, tea brown blight, and tea grey blight. A total of 1091 raw images of tea leaves affected by algal leaf spots, tea brown blight, and tea grey blight were captured at Wenhua Tea Farm, Miaoli City, Taiwan. For comparison, detection is also performed using the YOLOv3 Tiny, YOLOv3, YOLOv5, YOLOv8, and YOLOv10 models.

## 2. Data Acquisition

**Figure 1:**
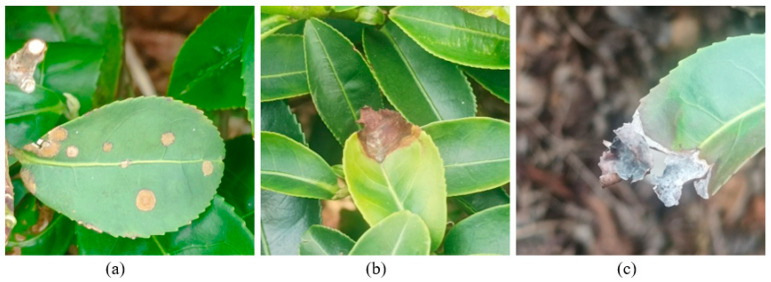
In situ photographs of tea leaves with (**a**) algal leaf spots, (**b**) brown blight, and (**c**) grey blight.

A total of 1091 raw images of tea leaves affected by algal leaf spots, tea brown blight, and tea grey blight were captured at Wenhua Tea Farm, Miaoli City, Taiwan. The images were obtained manually using a mobile phone (iPhone 11, Apple Inc, Cupertino, CA, USA) and had a size of 3024 × 4032 pixels, a bit depth of 24, and a resolution of 72 dpi. To maintain consistency and reliability, a meticulous camera protocol was adhered. Images were acquired from a standardized distance of approximately 0.5 m, ensuring minimal distortion and maintaining consistent object proportions. As shown in Figure 1, the leaves with algal leaf spot disease showed orange, flat spots with a furry texture. Depending on the severity, the disease manifested as either single spots or multiple spots. The leaves with brown blight exhibited small yellowish regions, which gradually changed to dark brown lesions as the disease progressed. The leaves with grey blight were similar to those with brown blight but displayed a white-greyish diseased region rather than a yellowish one. The image data is available at https://www.kaggle.com/datasets/yehyichang/tea-leaf-diseases (accessed on 18 August 2025).

**Figure 2:**
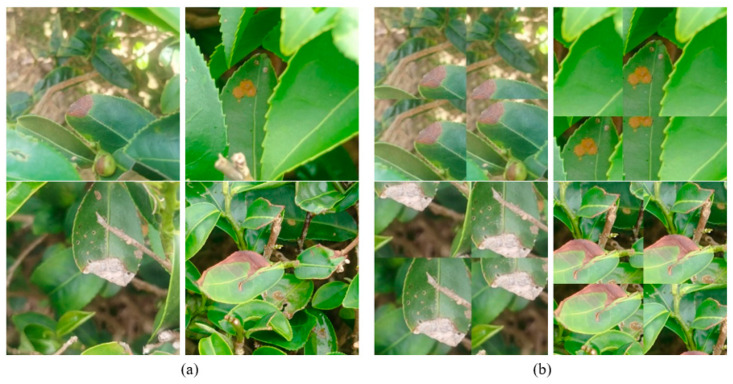
(**a**) Original images and (**b**) corresponding images after mosaic augmentation.

The dataset was preprocessed using mosaic augmentation to increase its size, thereby improving the robustness and generalization ability of the model [24]. In mosaic augmentation, four images are randomly selected, resized, cropped, and combined as a single image. After this, four bounding boxes are placed in one mosaic augmented image. After the crop is resized, the remaining part is used to generate another mosaic image if it contains bounding boxes; otherwise, it is removed. This augmentation helps to detect smaller objects because it generates four times more training data, prepares training data with different combined features that were never together in the same image to enhance learning capacity and diversity, and reduces the batch size by four times. Figure 2 compares four images before and after augmentation, respectively. During the augmentation process, the original images were cut into four patches, which were then randomly resized and stitched back together to create new composite images of the same size as the original image. The raw images were labelled using Roboflow software (https://roboflow.com/). It is noted that algal leaf spot was grouping multiple symptoms together and labelling as a bounding box. This method reduced the labelling time and provided higher detection accuracy. As shown in Table 1, the dataset was partitioned into a training set consisting of 2448 images and a test set comprising 275 images. Within each dataset, the number of instances of each disease class was approximately the same in order to avoid data imbalance. It is noted that the training set and test set of images are different.

## 3. Customized YOLOv5 Bi-HIC Model for Tea Leaf Disease Detection

**Figure 3:**
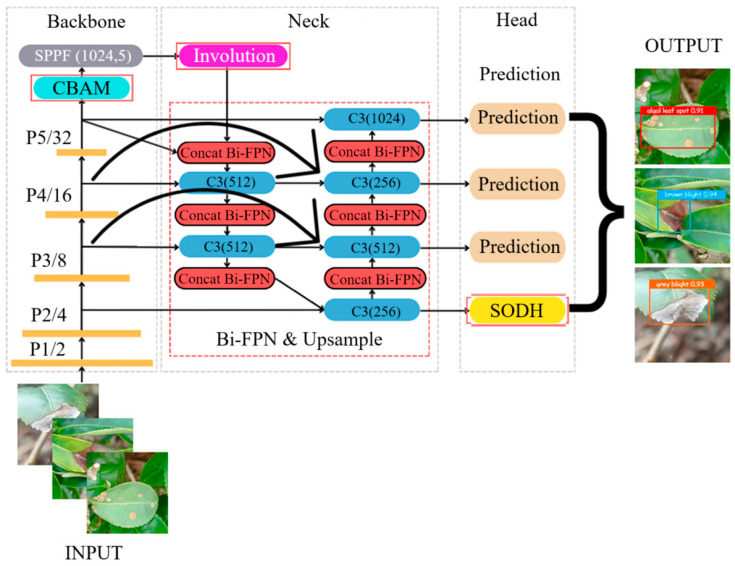
Basic architecture of YOLOv5 Bi-HIC model developed for tea leaf disease detection: a Bidirectional Feature Pyramid Network (Bi-FPN), a Small Object Detection Head (SODH), Involution, and Convolutional Block Attention Module (CBAM).

A customized model, designated as YOLOv5 Bi-HIC, was developed for tea leaf disease detection by adding four modules to the original YOLOv5 structure: a Bidirectional Feature Pyramid Network (Bi-FPN), a Small Object Detection Head (SODH), Involution, and Convolutional Block Attention Module (CBAM). The basic architecture of the proposed model is shown in Figure 3. The Bi-HIC model aims to boost the information flow through the Concat Bi-FPN module, which is integrated at the neck of the YOLOv5 backbone. The concatenation process fuses features from different layers or stages of the network, thereby allowing the model to leverage multiscale information more effectively. The Bi-FPN module enhances the feature fusion process by utilizing skip connections to yield better feature representations and improved detection performance. The SODH module serves as an additional prediction head in the YOLOv5 architecture to improve the prediction performance of the model for objects of varying sizes, particularly small objects. The CBAM is added at the end of the backbone to help the model focus on the most important features for detection. Finally, the Involution module is incorporated at the beginning of the neck to reduce the computational complexity.

In general, the Involution and CBAMse the detection of objects within complex backgrounds. Meanwhile, the additional prediction head, SODH, targets the detection of small-sized objects in the input image more effectively by utilizing higher-resolution feature maps to compensate for the smaller number of pixels representing the target objects in the image. By using a dedicated head for small objects, YOLOv5 Bi-HIC retains greater contextual information during the detection process and hence provides an improved performance in extracting the small features that are essential for distinguishing between different leaf diseases [25].

Involution aims to enhance the interaction between the channels and spatial locations in CNNs. The main purpose of involution is to reduce the computational complexity by minimizing the number of parameters while maintaining a high performance. Involution is designed to model long-range dependencies more effectively than traditional convolution, thereby improving the model performance [26,27]. Introduced by Li et al. [28], the key idea behind involution is to disperse the channel information to its spatial vicinity and vice versa by using a specialized kernel that operates on both the channel dimension and the spatial dimension simultaneously. This enables the use of a flexible receptive field size, which allows the global context to be captured more effectively and improves the ability of the model to extract features, particularly those of objects in complex backgrounds.

CBAM (Convolutional Block Attention Module) is an attention mechanism designed to improve the representational power of CNNs by focusing on significant features and suppressing less important ones. Attention mechanisms are particularly useful for extracting features from scenes in which details and precise localization are crucial for accurate detection. CBAM comprises two components: a Channel Attention Module (CAM) and a Spatial Attention Module (SAM). The CAM aims to capture the interdependencies between the channels in a convolutional layer, while the SAM captures the interdependencies between the spatial locations in the feature map. By combining the CAM and SAM, CBAM effectively captures both the channel-wise and the spatial-wise dependencies in the input feature maps, leading to improved feature representations that are useful for detecting objects in complex backgrounds [29].

**Figure 4:**
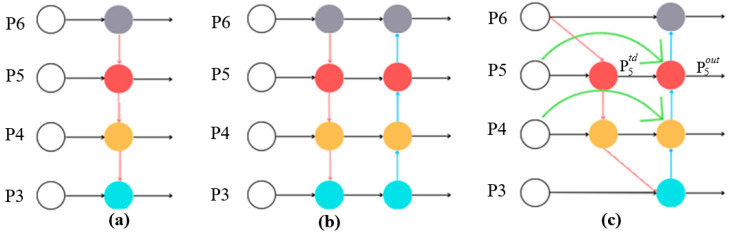
The schematic illustrates the main different of (**a**) FPN, (**b**) PANet, (**c**) Bi-FPN networks. FPNs utilize a top-down pathway to up sample the lower-resolution maps. PANet is an improved FPN that enhances the information flow in the feature pyramid by adding a bottom-up path. Bi-FPN optimizing the cross-scale connections in the network by removing nodes that have only one input edge and adding an additional edge from the original input to the output node if they are in the same layer.

The Bidirectional Feature Pyramid Network (Bi-FPN) is a feature pyramid architecture designed to efficiently fuse multi-scale features for object detection tasks. The primary objective of Bi-FPN is to enhance feature fusion by introducing bidirectional cross-scale connections and learnable weights. Bi-FPN is a refined version of the FPN and PANet (Path Aggregation Network) models. Figure 4 illustrates the main differences among the three structures. FPNs enhance the ability of a network to detect objects by leveraging information from multiple layers. FPNs utilize a top-down pathway to upsample the lower-resolution maps. The detailed features obtained from the higher-resolution maps are then added back to the lower-resolution maps [30]. FPN plays a key role in multiscale prediction; however, the information flow occurs only in the top-down direction. PANet is an improved FPN that enhances the information flow in the feature pyramid by adding a bottom-up path [31]. However, while PANet yields a significant improvement in the feature fusion process at different scales compared to previous versions of YOLO, it also increases the computational cost. Bi-FPN, presented by Tan et al. [32] seeks to address this problem by optimizing the cross-scale connections in the network by removing nodes that have only one input edge (i.e., minimal feature fusion) and adding an additional edge from the original input to the output node if they are in the same layer. Each bidirectional path is subsequently treated as a single feature network layer, where these layers can be stacked as needed to achieve higher-level feature fusion [33].

Mathematically, Bi-FPN is implemented as follows:

(1) $$P_{i}^{td}=Conv(\frac{\omega_{1}P_{i}^{in}+\omega_{2}Resize(P_{i+1}^{in})}{\omega_{1}+\omega_{2}+\varepsilon })$$

(2) $$P_{i}^{out}=Conv(\frac{\omega_{5}P_{i}^{td}+\omega_{6}P_{i}^{bu}}{\omega_{5}+\omega_{6}+\varepsilon })$$

where Conv is a convolution operation, Resize is *z* resizing (upsampling or downsampling) operation, $P_{i}^{in}$ is the input feature map of layer *i*, $P_{i}^{td}$ is the top-down feature map of layer i, $P_{i}^{out}$ is the final output feature map of layer i, ω is the learnable weight, and ε = 0.0001 is a small value that is used to avoid numerical instability. The bidirectional flow in Bi-FPN addresses challenges related to object-scale variations, reduces overlapping, and improves the ability of the model to capture the context [34]. In the present scenario, this additional context allows for the detection of tea leaf diseases characterized by spots and lesions of various sizes.

## 4. Results and Discussion

### 4.1. Evaluation Metric

The performance of the YOLOv5 Bi-HIC model was evaluated using the precision (P), recall (R), F1 score (F1), and mean average precision (mAP) metrics. Precision represents the ratio of the number of samples correctly predicted to be positive to the total number of positive predictions. The recall represents the ratio of the number of samples correctly predicted to be positive to the total number of positive samples (including both the true positives and the false negatives). The F1 score is the harmonic mean value of the precision and recall. Finally, the mAP indicates the accuracy (overlap) of the detected boundary boxes relative to the ground-truth bounding boxes. The metrics are obtained using a confusion matrix in accordance with the following formulas:

(3) $$Precision=\frac{TP}{TP+FP}$$

(4) $$Recall=\frac{TP}{TP+FN}$$

(5) $$F1=2\times \frac{Precision\times Recall}{Precision+Recall}$$

(6) $$mAP=\frac{1}{C}\sum_{k=i}^{N}P(k)\Delta R(k)$$

where True Positive (TP) is the number of positive samples that are correctly judged to be positive, False Positive (FP) is the number of negative samples judged to be positive, False Negative (FN) is the number of positive samples judged to be negative, and True Negative (TN) is the number of negative samples judged to be negative. In addition, P(k) represents the accuracy, and R(k) represents the recall. Training was conducted in Python 3.12 using a NVIDIA GeForce RTX 4070Ti GPU equipped with 4th-generation Tensor Cores. The GPU had 12 GB of GDDR6X memory (RAM) and 7680 CUDA cores to accelerate processing. The training parameters included the image size, batch size, and number of epochs. In accordance with the default settings of YOLOv5, the image size was set to 640 × 640. In addition, the batch size was set to 16, and 100 training epochs were performed to achieve an acceptable tradeoff between the computational accuracy and the runtime.

### 4.2. Experimental Setup and Results

Table 2 presents the training results for the four models. As shown, the proposed YOLOv5 Bi-HIC model achieved the highest precision (0.977), recall (0.943), mAP (0.968), and F1 Score (0.96) among all the models. By contrast, the YOLOv3 Tiny model yielded the lowest values of the four metrics (0.779, 0.831, 0.869, and 0.81, respectively). The YOLOv3 and YOLOv5 models achieved intermediate precision, recall, mAP, and F1 Score values of 0.896, 0.886, 0.916, and 0.89, and 0.951, 0.909, 0.950, and 0.93, respectively. The YOLOv8 and YOLOv10 models achieved higher precision, recall, mAP, and F1 Score values of 0.96, 0.93, 0.967, and 0.94, and 0.955, 0.917, 0.963, and 0.94, respectively. The superior precision of YOLOv5 Bi-HIC indicates a low rate of false positives, while the highest recall value demonstrates the ability of the model to detect nearly all the true positives. The enhanced detection performance of YOLOv5 Bi-HIC can be attributed in large part to the SODH module, which improves the ability of the model to detect small objects in the input image. For the images considered in the present study, the colour of the diseased regions is similar to that of the soil and lit regions in the background. These nuanced and complex details cannot be easily distinguished by the conventional YOLOv5 model, and hence, the model performance is degraded. However, the Bi-FPN and CBAMs proposed YOLOv5 Bi-HIC structure improve the ability of the model to detect and leverage contextual information in the input images. Consequently, the model consistently outperforms the other models across all the considered performance metrics.

**Figure 5:**
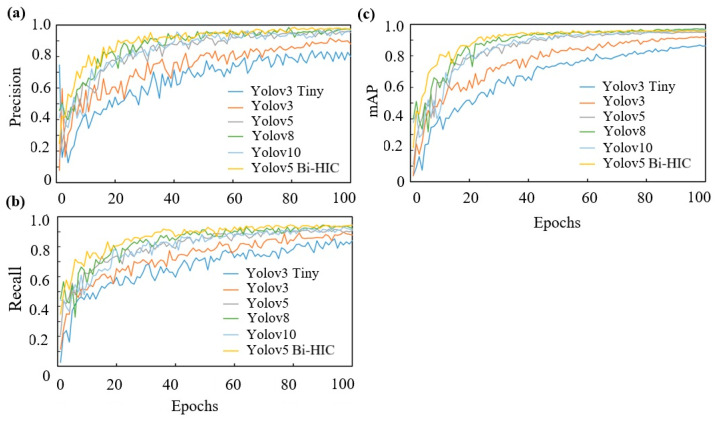
The training results of YOLO3-Tiny, YOLOv3, YOLOv5, YOLOv8, YOLOv10 and YOLOv5 Bi-HIC models (**a**) Precision, (**b**) Recall, (**c**) mAP.

Figure 5 shows the evolution of the precision, recall, mAP, and loss over the 100 epochs in the training process for the four models. It can be seen that the precision, recall, and mAP of the proposed YOLOv5 Bi-HIC model are not only the highest among all the considered models but also reach their maximum values more quickly. The precision curves show the greatest difference between the highest- and lowest-performing models. The YOLOv3 Tiny model exhibits the smallest improvement in the precision, recall, and mAP performance over the training process. This suggests that the smaller number of parameters and lower depth of the model structure inhibit its ability to extract all the necessary features, thereby resulting in a greater number of missed detections. YOLOv3 shows an improved precision compared to YOLOv3 Tiny, but its performance metrics still fail to surpass 0.9 by the end of the training process. The precision values of YOLOv5, YOLOv8, YOLOv10, and YOLOv5 Bi-HIC stabilize at high and similar values of 0.95 and 0.97, respectively. However, the precision of YOLOv5 Bi-HIC stabilizes after just 20–30 epochs, whereas that of YOLOv5 requires approximately 40 epochs to converge. For the YOLOv5, YOLOv8, YOLOv10, and YOLOv5 Bi-HIC models, the recall values are similar to but slightly lower than the precision values. By contrast, for the YOLOv3 Tiny model, the recall is slightly higher than the precision, which indicates that while it successfully identifies a large portion of the actual positive instances, it also generates a large number of false positives. The YOLOv5, YOLOv8, and YOLOv10 models both exhibit a better recall performance than the YOLOv3 Tiny and YOLOv3 models, with final values of at least 0.9. However, the recall performance of YOLOv5 Bi-HIC (0.94) is not only the highest among the four models but is also achieved more rapidly (after just 50 epochs).

In practice, the precision and recall metrics counteract each other. That is, as the precision increases, the recall decreases, and vice versa. Thus, the higher values of both metrics for YOLOv5 Bi-HIC compared to those of the base models indicate that it acquires a greater amount of detailed information and hence achieves an improved prediction performance. Higher precision and recall values lead to higher mAP values. YOLOv3 Tiny and YOLOv3 have lower depths than the other models and thus have the lowest mAP values over the course of the training process. YOLOv5 shows a much-improved performance and achieves a final mAP value of approximately 0.95. However, YOLOv5 Bi-HIC achieves an even higher mAP of approximately 0.968.

The four models show a very low loss during the training process, which indicates that they all perform well in correctly predicting the different classes. YOLOv3 Tiny has a slightly higher loss (0.002) than the other models. In addition, the losses of the YOLOv3 Tiny and YOLOv3 models are relatively unstable and show slight evidence of overfitting, as indicated by a slight upward curve of the corresponding profiles after the validation stage. Notably, such a tendency is not observed for the YOLOv5 Bi-HIC architecture, which demonstrates the effectiveness of the additional modules in extracting the detailed features and contextual information required to extract the target objects properly.

**Figure 6:**
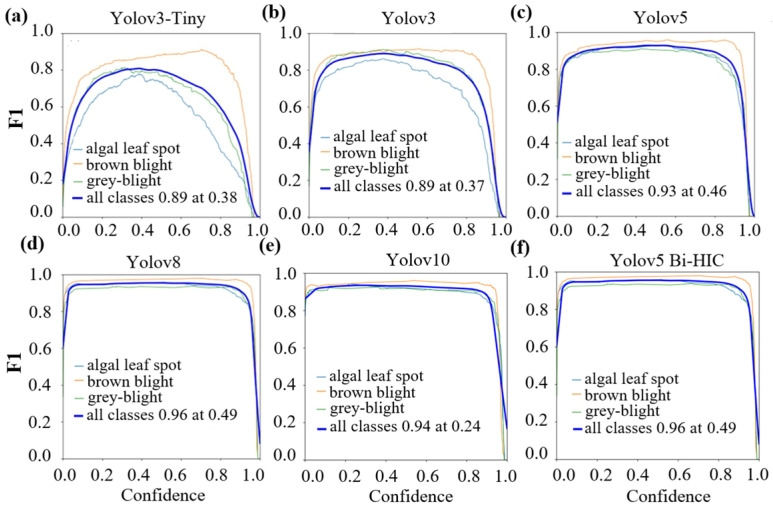
F1-Score curves for each disease using different models: (**a**) YOLOv3 Tiny, (**b**) YOLOv3, (**c**) YOLOv5, (**d**) YOLOv8, (**e**) YOLOv10 and (**f**) YOLOv5 Bi-HIC.

Figure 6 shows the F1 scores of the four models for the three leaf diseases. A larger area under the curve (AUC) indicates an improved detection performance of the model for the corresponding disease and hence a higher F1 score. The curves of the YOLOv3 Tiny model have a broad peak-like characteristic in which the F1 score first increases gradually with an increasing confidence value and then decreases. Moreover, the curves associated with the different diseases are widely dispersed. In other words, the model shows a nonuniform classification accuracy across the different diseases. The AUCs are also relatively small, indicating a poor detection performance across the four diseases. YOLOv3 shows larger AUCs for all the diseases. Moreover, the peak value of the F1 score (0.89) extends over a broader confidence value range (0.2–0.4). However, the F1 score remains below 0.9 and decreases at higher confidence levels, particularly for the algal leaf spot disease. YOLOv5, YOLOv8, and YOLOv10 show a high peak F1 value of 0.93~0.96 over an extended confidence range of 0.1–0.7. However, the F1 value decreases at confidence levels over 0.8. The proposed YOLOv5 Bi-HIC model shows a larger AUC than the other models and thus provides better confidence in predicting the different diseases. Moreover, the peak F1 score (0.96) spans an expanded confidence level range of 0.1–0.9. In addition, the F1 score curves for the different diseases are very close to each other. In other words, the YOLOv5 Bi-HIC model has a similar classification performance for all four diseases.

For all the models, the brown blight class yields the largest AUC and is the most reliably detected disease among the three. For the YOLOv3 Tiny and YOLOv3 models, the detection performance for the algal leaf spot disease is significantly reduced compared to that for the brown and grey blight diseases. With more layers, the YOLOv5 models achieve a higher and more consistent detection performance across the three diseases than the YOLOv3 models. Among the four models, the optimal detection performance is achieved using the proposed YOLOv5 Bi-HIC model due to the combined effects of the four modules in extracting a greater amount of detailed and contextual information.

**Figure 7:**
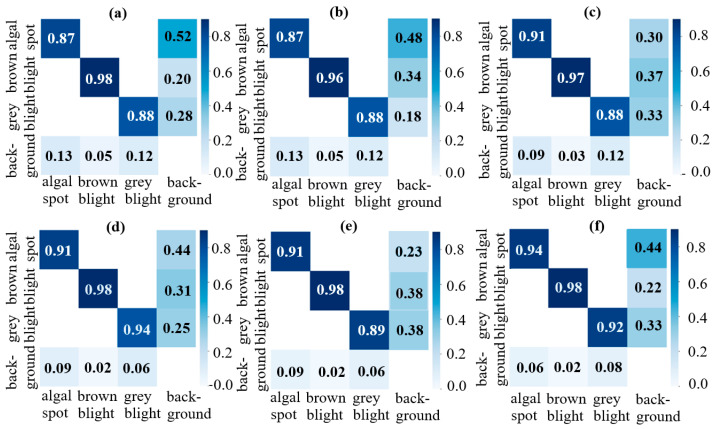
Confusion matrices for (**a**) YOLOv3 Tiny, (**b**) YOLOv3, (**c**) YOLOv5, (**d**) YOLOv8, (**e**) YOLOv10 and (**f**) YOLOv5 Bi-HIC models.

Figure 7 shows the confusion matrices of the four models. For each matrix, the boxes along the diagonal indicate the correct classification of the corresponding object to its class. In contrast, the other boxes indicate false classifications of the corresponding object against the background. In general, algal leaf spot disease is complex to classify correctly because the spots are small, and hence the model requires deeper and more strongly discriminating extracting layers. Observing the four classification matrices, YOLOv3 Tiny achieves the poorest detection performance among the four models. In particular, it achieves classification accuracies of 0.86, 0.95, and 0.88 for the algal leaf spot, brown blight, and grey blight diseases, respectively. The poor performance of YOLOv3 Tiny can be attributed to its simple structure and shallow depth, which limit its ability to extract contextual and target-relevant features. YOLOv3 achieves slightly improved detection accuracies of 0.87, 0.96, and 0.88 for the algal leaf spot, brown blight, and grey blight diseases, respectively, but the lack of layers still constrains its performance. YOLOv5 achieves a detection accuracy higher than 90% for the algal leaf spot (0.91) and brown blight (0.96) diseases. YOLOv8 and YOLOv10 achieve a detection accuracy higher than 90% for the algal leaf spot (0.91) and brown blight (0.98) diseases. However, YOLOv10 has a lower accuracy of 0.89 for the grey blight disease since, in some cases, the diseased region is overexposed due to lighting effects or is occluded by other leaves. The lighting makes the leaves reflect white and become similar to the grey blight symptoms. The detection accuracies for the YOLOv5 Bi-HIC model have high values of 0.94 for algal leaf spot, 0.98 for brown blight, and 0.92 for grey blight. In other words, the additional modules enhance the ability of the model to perform object detection, even for small-sized objects such as those that characterize algal lead spot disease, despite the presence of complex backgrounds. Notably, the detection accuracies of YOLOv5 Bi-HIC are not only higher than those of the other models but are also more closely aligned across the three diseases. In other words, YOLOv5 Bi-HIC achieves a higher detection accuracy than the other models and a superior robustness across the different diseases.

**Figure 8:**
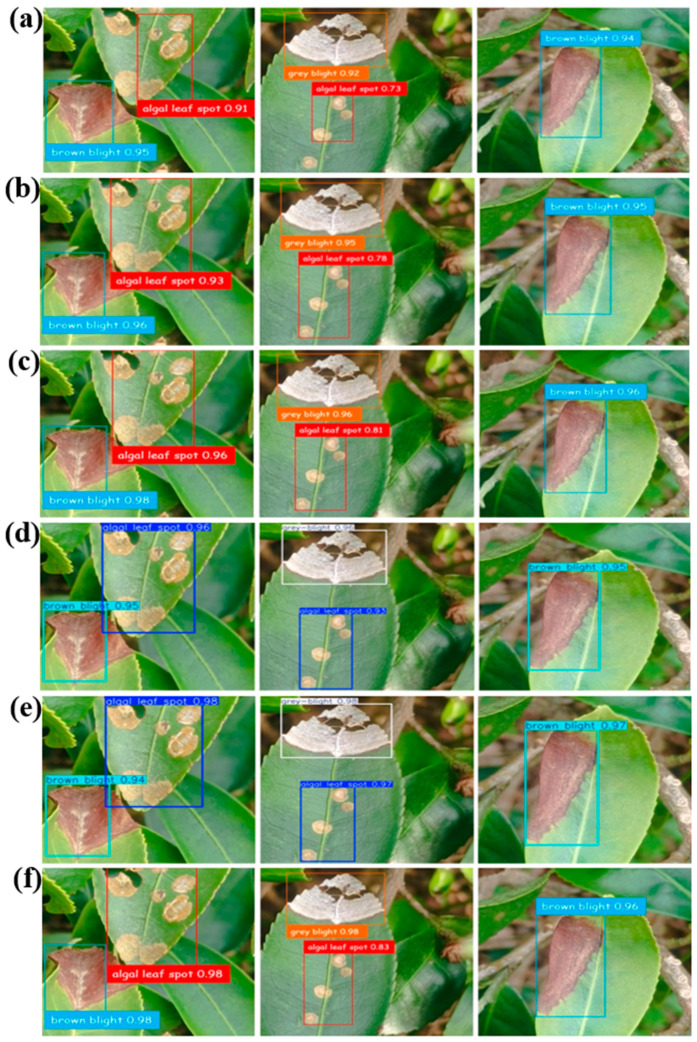
Typical detection results obtained using (**a**) YOLOv3 Tiny, (**b**) YOLOv3, (**c**) YOLOv5, (**d**) YOLOv8, (**e**) YOLOv10 and (**f**) YOLOv5 Bi-HIC models for three tea leaf diseases.

Figure 8 shows typical detection results of the four models for the three diseases. YOLOv3 Tiny shows a confidence score of over 0.9 for detecting brown blight (0.94 and 0.95) and grey blight (0.92). However, its confidence level for detecting algal leaf spot disease is only 0.73. In addition, the model fails to detect some spots on the leaf as a result of its inability to extract all the salient features during training. YOLOv3 yields higher confidence scores for brown blight (0.95 and 0.96) and grey blight (0.95), successfully identifying more spots associated with algal leaf spot disease. However, it still achieves a low confidence score of only 0.78. YOLOv5 achieves a similar detection performance to YOLOv3, but with higher confidence scores of 0.96 and 0.98 for brown blight, 0.96 for grey blight, and 0.81 for algal leaf spot, due to its deeper layers and increased connections between them. YOLOv8 yields higher confidence scores for brown bright (0.95) and successfully identifies more spots associated with algal leaf spot disease (0.96 and 0.93). YOLOv10 yields higher confidence scores for algal leaf spot (0.98 and 0.97) and successfully identifies more spots associated with brown blight disease (0.97 and 0.94). The YOLOv5 Bi-HIC model shows a further improvement in the detection confidence, with scores of 0.96 and 0.98 for brown blight, 0.98 for grey blight, and 0.83 for algal leaf spot. In addition, the bounding boxes accurately enclose the diseased areas, which is crucial for precise localization and further analysis or intervention. The proposed technique can generally detect tea leaf disease with the highest accuracy of 98%. The detection accuracy is higher than that proposed in Ref. [34] using Yolov8 for tea leaf detection with an accuracy of 89%, or Ref. [21] by using YOLOv8-ASFF with an accuracy of 95%. Moreover, the proposed model accuracy is compatible with that proposed in Ref. [35] by using the YOLOv8-RCAA model with a detection accuracy of 98% or Ref. [36] by using YOLOv10-Powered for tea leaf disease detection with an accuracy of 98.9%. In general, the proposed YOLOv5 Bi-HIC model provides an efficient method for tea leaf disease detection in large-scale tea farms with high accuracy, less time-consuming, and cost-saving.

## 5. Conclusions

This study has presented an enhanced CNN model named YOLOv5 Bi-HIC for detecting three tea leaf diseases: algal leaf spot, tea brown blight, and tea grey blight. Compared to the traditional YOLOv5 model, YOLOv5 Bi-HIC incorporates a new prediction head (SODH) to improve the detection performance for small objects, an Involution module to reduce the computational complexity, an attention module (CBAM) to help the model focus on the extraction of important features, and a Bi-FPN module to enhance multiscale prediction and boost information flow, thereby extracting more details and important features during the detection process. The detection performance of YOLOv5 Bi-HIC has been compared with that of three other YOLO models (YOLOv3 Tiny, YOLOv3, and YOLOv5) using a total of 2723 images containing multiple instances of the three tea leaf diseases. YOLOv5 Bi-HIC achieved precision, recall, mAP, and F1 scores of 0.977, 0.943, 0.968, and 0.96, respectively, during training. The performance of the proposed model surpassed that of the other three models. Furthermore, YOLOv5 Bi-HIC showed a lower tendency to falsely classify target objects as background, which is particularly important in tea leaf disease detection, in which the target objects are small and the backgrounds are complex. Overall, the architectural enhancements implemented in YOLOv5 Bi-HIC facilitate extracting detailed and critical information from the input images, mitigating overfitting and ensuring a low and stable classification loss during training. The results indicate that YOLOv5 Bi-HIC provides a proficient, high-precision, and high-recall approach for the in situ detection of tea leaf diseases in tea plantations, thereby enabling timely and effective intervention strategies to improve the tea yield and quality.

## Figures and Tables

**Table 1:** Tea leaf disease datasets.

Diseases	Training Set	Test Set
Algal leaf spot	839	94
Tea brown blight	803	90
Tea grey blight	806	91
Total	2448	275

**Table 2:** Training Results.

Model	Precision (P)	Recall (R)	mAP	F1 Score
YOLOv3 Tiny	0.779	0.831	0.869	0.81
YOLOv3	0.896	0.886	0.916	0.89
YOLOv5	0.951	0.909	0.950	0.93
YOLOv8	0.960	0.930	0.967	0.94
YOLOv10	0.955	0.917	0.963	0.94
YOLOv5 Bi-HIC	0.977	0.943	0.968	0.96

## Data Availability

The original contributions presented in this study are included in the article. Further inquiries can be directed to the corresponding authors.
